# Impact of COVID-19 on Respiratory Function: A Post-Recovery Comparative Assessment

**DOI:** 10.3390/jcm15020717

**Published:** 2026-01-15

**Authors:** Daniela Robu Popa, Corina Marginean, Mona Elisabeta Dobrin, Radu Adrian Crisan Dabija, Oana-Elena Melinte, Stefan Dumitrache-Rujinski, Ioan Emanuel Stavarache, Ionel-Bogdan Cioroiu, Antigona Carmen Trofor

**Affiliations:** 1 “Grigore T. Popa” University of Medicine and Pharmacy, 700115 Iasi, Romania; daniela.robu-popa@d.umfiasi.ro (D.R.P.); radu.dabija@umfiasi.ro (R.A.C.D.); oana-elena.melinte@umfiasi.ro (O.-E.M.); stavarash@yahoo.com (I.E.S.); antigona.trofor@umfiasi.ro (A.C.T.); 2 Oncology and Palliative Care Department, “George Emil Palade” University of Medicine and Pharmacy, Science and Technology of Târgu Mureș, 540142 Târgu Mureș, Romania; 3 Biochemistry Department, Clinical Hospital of Pulmonary Diseases, 700116 Iasi, Romania; elisabeta-mona.dobrin@pneumo-iasi.ro; 4 Department of Pneumophtysiology I, “Carol Davila” University of Medicine and Pharmacy, Dionisie Lupu Street, Nr. 37, Sector 2, 020021 Bucharest, Romania; stefan.dumitrache@umfcd.ro; 5 Department of Preclinical Disciplines, Faculty of Medicine, “Apollonia” University of Iaşi, 700511 Iași, Romania; 6 Romanian Academy-Iasi Branch, Research Center for Oenology, 700490 Iasi, Romania; bogdan.cioroiu@acadiasi.ro

**Keywords:** post-COVID-19 syndrome, respiratory function tests, DLCO

## Abstract

**Background**: Post-COVID-19 syndrome (PCS) is defined as the persistence or development of new symptoms 3 months after the initial infection with the SARS-CoV-2 virus, these clinical aspects being most often associated with functional respiratory changes, as well as imagistic modifications. This study aimed to evaluate longitudinal changes in pulmonary function among patients with PCS, in relation to the severity of the acute COVID-19 episode and the time elapsed since infection. **Methods**: A retrospective, observational study was conducted at the Clinical Hospital of Pulmonary Diseases Iași, Romania, between January 2021 and December 2022, including 97 adult patients with confirmed PCS. Demographic, clinical, and functional data were collected from medical records. Pulmonary function tests (PFTs) were performed according to ATS/ERS standards, assessing Forced Vital Capacity (FVC), Forced Expiratory Volume in the First Second (FEV_1_), FEV_1_/FVC ratio (Tiffeneau Index), Maximal Expiratory Flow at 50% and 25% of FVC (MEF50, MEF25), Diffusing Capacity of the Lung for Carbon Monoxide (adjusted for haemoglobin) (DLCO), Carbon Monoxide Transfer Coefficient (KCO), Alveolar Volume (AV), Total Lung Capacity (TLC) and Residual Volume (RV). Patients were grouped by time elapsed since infection (1–3, 4–7, 9–12, and up to 22 months). Statistical analyses included the Mann–Whitney U test, Spearman’s correlation, ROC curve analysis, and Principal Component Analysis (PCA). **Results**: A progressive improvement in FVC was observed up to 9–18 months post-infection (*p* < 0.05), while FEV_1_ remained stable, suggesting a predominantly restrictive ventilatory pattern. Patients with moderate acute COVID-19 presented significantly lower FVC%, FEV_1_%, DLCO%, and KCO% values compared with those with mild disease (*p* < 0.05). Diffusion abnormalities (DLCO and KCO) persisted beyond 12 months, indicating lasting alveolar-capillary impairment. ROC analysis identified TLC (AUC = 0.857), AV (AUC = 0.855), and KCO (AUC = 0.805) as the most discriminative parameters for residual dysfunction. PCA revealed three major functional domains—airflow limitation, diffusion capacity, and lung volume—explaining up to 70% of total variance. **Conclusions**: We are facing the emergence of a new phenomenon, namely a secondary post-COVID-19 pandemic of patients confronting with persistent post-COVID-19 symptoms who present with functional respiratory changes and who require careful monitoring in dynamics, personalized treatments and a multidisciplinary approach.

## 1. Introduction

Since December 2019, the entire globe has been confronted with the most dramatic and frightening experience of the last century, the COVID-19 (Coronavirus Disease 2019) pandemic [1].

According to the World Health Organization (WHO), there have been over 778 million cases globally, of which over 7 million have resulted in deaths. In Romania, there have been over 3.5 million cases, of which over 68,000 have resulted in deaths [2].

In terms of clinical aspects, the disease can vary considerably, from completely asymptomatic forms, to forms with general, nonspecific symptoms, to severe ones, with acute respiratory failure and multiple organ dysfunction. Among the most common complications cited in the literature are viral pneumonia, bacterial or fungal infections, septic shock, acute respiratory distress syndrome, arrhythmias, acute coronary syndrome, myocarditis, renal failure, thromboembolic or hemorrhagic complications, gastrointestinal or neurological complications [3,4].

While some patients recover during the post-acute phase (within the first four weeks), it appears that over half of them present symptoms at 3 months after the acute viral episode, a phenomenon known as chronic post-COVID-19 syndrome [5,6,7].

Post-COVID-19 syndrome is defined as the persistence or development of new symptoms 3 months after the initial infection with the SARS-CoV-2 virus, these symptoms lasting at least 2 months and without being associated with other pathology [8]. To date, no standardized criteria have been established for the precise definition of post-COVID-19 syndrome (PCS). Clinicians face a wide spectrum of symptoms, accompanied by paraclinical, imaging, and respiratory functional alterations, in patients presenting for follow-up after the acute phase of COVID-19 [5,6].

Specialists can quantify clinical, paraclinical, imaging and respiratory functional changes that persist beyond the acute viral episode and that require additional investigations and referral of patients to centers specialized in the evaluation and management of post-COVID-19 pathology [9].

Conducting rigorous follow-up among patients with post-COVID-19 syndrome and early identification of risk factors associated with an unfavorable prognosis are crucial in improving patient outcomes. Among the most important risk factors are old age and the presence of comorbidities. Cardiovascular disease, chronic respiratory disease, metabolic syndrome, immunosuppressive conditions (human immunodeficiency virus infection, neoplasia, organ transplantation), autoimmune disease, chronic kidney disease, liver disease, neurological disease, as well as nicotine and alcohol dependence are among the most frequently cited conditions associated with the occurrence of moderate and severe forms of COVID-19 disease and subsequently of post-COVID-19 syndrome [10,11,12,13].

The lung is the organ most affected by the SARS-CoV-2 virus, and patients with post-COVID-19 status may present with dynamic respiratory functional impairment. Pulmonary function assessment involves performing several investigations such as spirometry, measurement of gas transfer through the alveolar-capillary membrane (TLCO or DLCO), or body-plethysmography [6].

The most important functional changes observed among patients with post-COVID-19 syndrome are the following: impairment of pulmonary diffusion function (proven by a decrease in DLCO), restrictive ventilatory syndrome with increased pulmonary elastic recoil, spirometrically materialized by decreases in forced vital capacity and increases in the Tiffeneau index, aspects that may suggest a certain degree of post-COVID-19 fibrosis, as well as obstructive ventilatory syndrome (materialized by a decrease in the Tiffeneau index) [14,15].

The most common respiratory functional change cited in the literature among post-COVID-19 patients is impaired pulmonary diffusion capacity (decreased DLCO), followed by decreased Total Lung Capacity (TLC), Forced Vital Capacity (FVC), and Forced Expiratory Volume in 1 Second (FEV_1_), these changes being directly proportional to the severity of the disease in the acute phase [16,17].

It seems that this category of patients present persistence or the appearance of symptoms such as physical asthenia, dyspnea or cough, associated with functional respiratory changes and imagistic abnormalities [18,19].

In addition, recent studies have shown increased addressability even 1–2 years on from infection, especially among patients who have developed moderate–severe forms of COVID-19, which underlines the imperative need for rigorous monitoring of the evolution of this condition in order to identify any complications that may arise and to minimize long-term effects [19,20].

The aim of the present study is to evaluate changes in lung function in patients who have suffered from the COVID-19 disease and to correlate the type and magnitude of lung function deficit with the clinical form and the severity of COVID-19. In addition, we analyzed the persistence and long-term fluctuations (over 6, 12 months) of clinical and functional changes and the associated risk factors. Also, we analyzed the comparative evolution of lung function parameters in patients evaluated in 2021 and 2022 in order to identify significant changes in the severity or profile of post-COVID-19 lung damage.

## 2. Materials and Methods

This retrospective, observational, analytical study was conducted between January 2021 and December 2022 at the Clinical Hospital of Pulmonary Diseases Iasi, a regional reference center for the diagnosis and management of pulmonary diseases in northeastern Romania.

### 2.1. Study Population

A total of 97 adult patients with a history of confirmed SARS-CoV-2 infection, subsequently diagnosed with PCS, were included. Diagnosis of both acute COVID-19 and post-COVID-19 syndrome (PCS) was established based on the criteria outlined by the World Health Organization guidelines for the diagnosis and management of COVID-19 [21]. Only patients with no documented history of pulmonary impairment prior to the COVID-19 episode were included in the study. They presented to the hospital for further investigations, having either developed or persisted with symptoms that formed the basis for the diagnosis of post-COVID syndrome.

Patient selection was carried out to include, as much as possible, individuals without significant respiratory comorbidities, with the aim of minimizing the influence of pre-existing pulmonary diseases on the results of lung function tests. By excluding the cases with chronic respiratory conditions, the analysis sought to most accurately reflect the specific impact of SARS-CoV-2 infection on lung function over time. Among the reported comorbidities, hypertension was observed in 14–36% of patients, followed by diabetes mellitus (5–20%), cardiovascular disease (14%), neoplastic disorders (10%), thyroid disease (11%), and rheumatologic diseases (11%) (Table 1 and Table 2).

Inclusion Criteria:

- Age ≥ 18 years
- Confirmed diagnosis of post-COVID-19 syndrome
- Availability for pulmonary function testing
- Signed informed consent

Exclusion Criteria:

- Age < 18 years
- Absence of informed consent
- Pregnancy or breastfeeding
- Bronchial asthma
- Interstitial lung diseases (including sarcoidosis and pulmonary fibrosis)
- Chronic obstructive pulmonary disease (COPD)
- Bronchiectasis
- Active respiratory infection within 4–6 weeks prior to assessment
- Active or recent pulmonary malignancy

### 2.2. Data Collection

Demographic and clinical data were extracted from electronic and paper medical records. Collected variables included: age, sex, place of residence (urban/rural), and smoking history. Clinical information such as oxygen saturation (SpO_2_) at the time of evaluation, persistent post-COVID-19 symptoms, and pre-existing comorbidities were also recorded. Persistent post-COVID symptoms, including physical fatigue, dyspnea, and cough, were recorded across all follow-up intervals, with variable prevalence between groups.

### 2.3. Pulmonary Function Testing

All patients underwent standardized pulmonary function testing (PFT) performed in the Laboratory of Pulmonary Function Test of the hospital, using MasterScreen Body and CareFusion devices (JAEGER Medical GmbH, Hochberg, Germany). Tests were conducted in accordance with the recommendations of the American Thoracic Society (ATS) and the European Respiratory Society (ERS) [22].

The following parameters were measured and analyzed: FVC: Forced Vital Capacity, FEV_1_: Forced Expiratory Volume in the First Second, FEV_1_/FVC ratio (Tiffeneau Index), MEF50, MEF25: Maximal Expiratory Flow at 50% and 25% of FVC, DLCO: Diffusing Capacity of the Lung for Carbon Monoxide (adjusted for haemoglobin), KCO: Carbon Monoxide Transfer Coefficient, AV: Alveolar Volume, TLC: Total Lung Capacity, RV: Residual Volume. Quality control procedures, including routine calibration of equipment, were rigorously followed in accordance with laboratory protocols.

### 2.4. Patient Grouping Based on Time Since Infection

To assess the progression of lung function over time, patients were categorized based on the interval between the acute COVID-19 episode and the date of pulmonary function testing. Patients evaluated in 2021 (*n* = 65) were grouped as follows: 1–3 months post-COVID-19, 4–7 months post-COVID-19, 9–12 months post-COVID-19. Patients evaluated in 2022 (*n* = 32) were grouped as follows: 3–6 months post-COVID-19, 7–12 months post-COVID-19, 14–22 months post-COVID-19. This stratification enabled longitudinal analysis of pulmonary recovery, allowing comparison of functional parameters over different post-infection intervals, as well as exploring of associations with disease severity and comorbidities.

### 2.5. Statistical Analysis

Descriptive statistical analysis of the demographic and clinical characteristics of the patients included in the study was performed using STATISTICA 10 and R software (version 4.5.1), by calculating the mean, standard deviation, minimum, and maximum values. These variables were interpreted in relation to the occurrence of acute episodes among patients with post-COVID-19 syndrome. Differences between the study groups—patients with mild, moderate, and severe forms of the disease—were assessed using the non-parametric Mann–Whitney U test, due to the non-normal distribution of the data. Spearman’s rank correlation coefficient was applied to evaluate correlations between clinical parameters and to identify potential risk factors associated with disease severity. A *p*-value of < 0.05 was considered statistically significant for all analyses. Factorial analysis (FA) was performed to identify latent variables associated with lung function assessment and gas exchange efficiency in patients diagnosed with post-COVID-19 syndrome. To assess the diagnostic performance of each clinical parameter, Receiver Operating Characteristic (ROC) curves were generated, and the Area Under the Curve (AUC) was calculated.

### 2.6. Ethics Consideration

This study was approved by the ethics committee of the Clinical Hospital of Pulmonary Diseases, Iasi, Romania (ethical approval no. 98/16 March 2023) and by the ethics committee of the University of Medicine and Pharmacy “Grigore T. Popa” Iasi, Romania (ethical approval no 317/29 May 2023).

## 3. Results

### 3.1. Study Population and Baseline Characteristics

Pulmonary function was assessed in three groups of patients with post-COVID-19 syndrome evaluated at 1–3 months (*n* = 36), 4–7 months (*n* = 18), and 9–12 months (*n* = 11) after the acute viral episode. The mean age was similar among groups (52.2 ± 12.73, 54.78 ± 15.79, and 53.5 ± 17.31 years, respectively; no significant differences). Participants were predominantly from urban areas, comprising 86% of the 1–3-month group, 70% of the 4–7-month group, and 90% of the 9–12-month group. Female participants accounted for 61%, 55%, and 70% of the respective groups. Severe acute COVID-19 was present exclusively in the 4–7-month group (10%), while no cases were identified in the 1–3-month or 9–12-month groups, resulting in a significant difference across groups. (Table 1).

A progressive increase in the prevalence of comorbidities was observed over time, particularly for arterial hypertension (11% at 1–3 months → 36% at 9–12 months), diabetes mellitus (5% → 18%), and obesity (5–10%), possibly due to different group selection.

Regarding post-COVID-19 symptoms, the most frequent complaint was physical asthenia, reported by 69% of patients at 1–3 months, decreasing to 40% at 4–7 months, and rising again to 60% at 9–12 months, possibly reflecting the severity of the initial disease. Dyspnea remained relatively stable over time (40–50%), while cough showed a slight peak in the 4–7-month group (35%) (Table 1).

Pulmonary function analysis revealed that at 9–12 months after the acute episode, patients showed significant reductions in FVC, FEV_1_, and DLCO (Table 1), suggesting persistent pulmonary impairment, particularly among those with severe acute disease.

In the cohort investigated in 2022, 32 patients were included and divided into three groups according to the time since acute infection: Group 1 (PCS onset at 3–6 months, *n* = 19), Group 2 (PCS onset at 7–12 months, *n* = 6), and Group 3 (PCS onset at 14–22 months, *n* = 7). (Table 2).

Demographic data, comorbidities, clinical form of the disease, persistent symptoms, and respiratory functional parameters (FVC, FEV_1_, FEV_1_/FVC, DLCO, KCO, MEF 25/50%, and peripheral oxygen saturation—SpO_2_) were collected.

Descriptive statistics identified dyspnea as the most frequent persistent symptom in Group 3 (60%), while physical asthenia predominated in earlier phases (45%) (Table 2). Spirometric parameters (FVC and FEV_1_) generally remained within or close to normal ranges, with a slight decreasing trend in the group with PCS onset more than 12 months after the acute viral episode. MEF 25/50% values were particularly reduced in Group 3 (14–22 months), suggesting small airway involvement. Mean DLCO values were significantly impaired across all three groups, with the lowest mean recorded in Group 3 (79.40%). Peripheral oxygen saturation remained generally normal, although some patients in Group 3 presented values below 88%. A higher frequency of cardiovascular and metabolic comorbidities was noted, with hypertension being consistently present, while the total number of comorbidities decreased after 6 months (Table 2).

**Figure 1:**
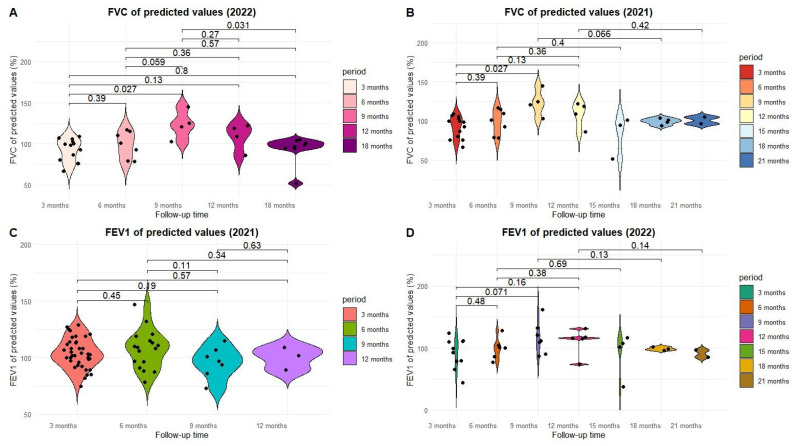
Evolution of post-COVID-19 pulmonary function: analysis of FVC (%) and FEV_1_ (%) parameters in patients evaluated during 2021–2022. (**A**) FVC of predicted values (2022), (**B**) FVC of predicted values (2021), (**C**) FEV1 of predicted values (2021), (**D**) FEV1 of predicted values (2022).

The study evaluated pulmonary function in patients with PCS assessed in 2021 and 2022, using FVC and FEV_1_ (expressed as percentages of predicted values), analyzed according to the time elapsed since the acute episode. The distribution of these values was graphically represented using violin plots (Figure 1), and showed individual variability and median trends across follow-up intervals.

Analysis of the evolution of pulmonary function parameters in PCS patients (Figure 1) revealed a progressive improvement in forced vital capacity (FVC) in both cohorts. In patients evaluated in 2021, FVC values increased between 3 and 9 months post-infection (*p* = 0.027), indicating clinically relevant functional recovery, followed by stabilization after 9 months. In the 2022 cohort, a similar trend was observed, with significant differences between the 3–9 month (*p* = 0.027) and 9–18 month (*p* = 0.031) intervals, suggesting prolonged pulmonary recovery up to 18 months post-infection [23].

In contrast, FEV_1_ values did not show significant changes in either year (*p* > 0.1), suggesting that the predominant ventilatory impairment is restrictive, with relatively preserved expiratory flows [24].

Differences between patients with mild and moderate COVID-19 were assessed using the Mann–Whitney U test (significance threshold: *p* < 0.05), which revealed no significant differences in SpO_2_, IT, MEF 50 (%), or MEF 25 (%) between the two groups (Table 3).

Significant differences were observed between patients with mild and moderate post-COVID-19 forms. In the 2021 assessment, subjects with moderate disease were older (*p* < 0.001) and showed significantly lower values of FVC% (*p* = 0.001), FEV_1_% (*p* = 0.019), DLCO% (*p* = 0.002), and KCO% (*p* = 0.002) compared with those with mild disease. At the 2022 follow-up, differences persisted for DLCO% (*p* = 0.001) and KCO% (*p* = 0.003), indicating a sustained impairment of alveolar-capillary diffusion capacity in patients with moderate COVID-19 severity (Table 3).

### 3.2. ROC Analysis of Pulmonary Function in Post-COVID-19 Patients (2021–2022)

**Figure 2:**
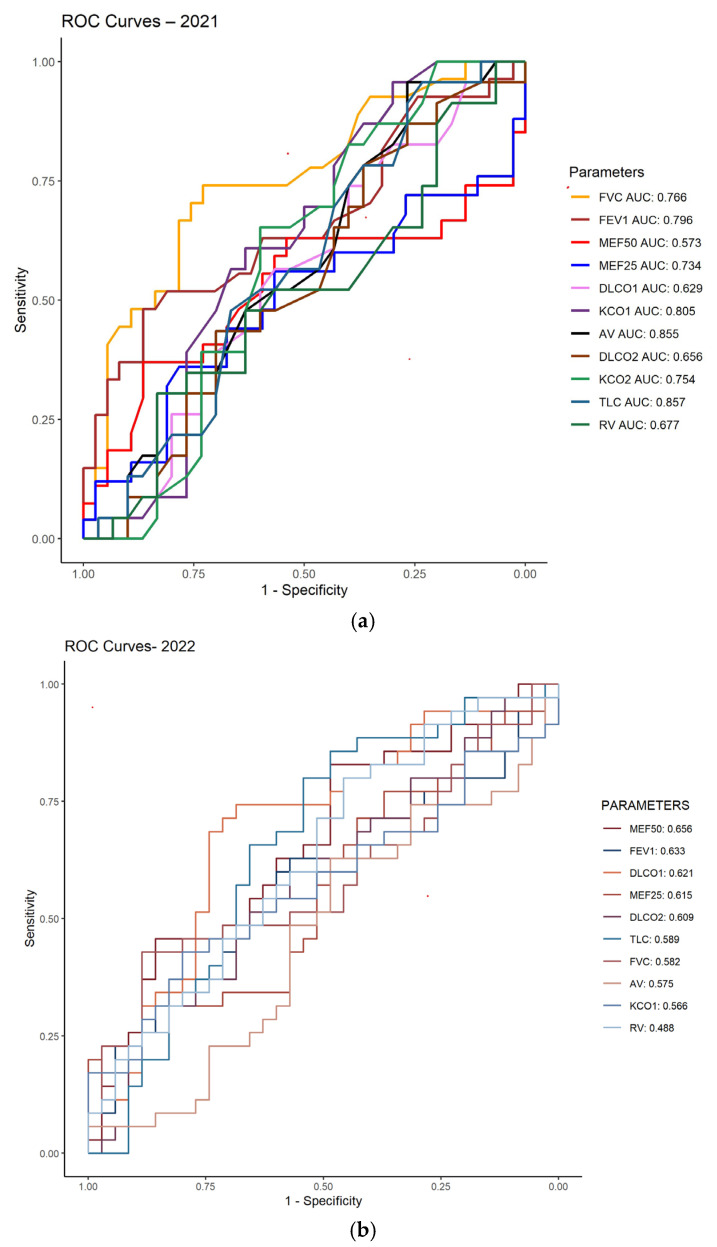
(**a**). ROC curves for pulmonary function parameters in patients diagnosed with post-COVID-19 syndrome in 2021. (**b**). ROC curves for pulmonary function parameters in patients diagnosed with post-COVID-19 syndrome in 2022.

To identify respiratory functional parameters with predictive value for pulmonary impairment in PCS patients, Receiver Operating Characteristic (ROC) curve analysis was performed. This method allowed the assessment of diagnostic performance of the evaluated variables by relating sensitivity and specificity across different decision thresholds. The area under the curve (AUC) was used as a global indicator of discriminative ability. The results are presented in Figure 2a,b.

Among the evaluated parameters, total lung capacity (TLC) showed the highest diagnostic performance (AUC = 0.857), followed by alveolar ventilation (AV, AUC = 0.855) and the carbon monoxide transfer coefficient relative to alveolar volume (KCO, AUC = 0.805). These three parameters demonstrated AUC values > 0.80, indicating high predictive capacity for post-COVID-19 pulmonary impairment. FVC and FEV_1_ exhibited AUC values of 0.796, corresponding to moderate diagnostic performance. In contrast, MEF50 (AUC = 0.573) and DLCO (AUC = 0.656) showed limited predictive ability, suggesting a lower relevance in identifying residual pulmonary impairment.

Regarding the performance of pulmonary function parameters in discriminating patients with post-COVID-19 impairment in 2022, MEF50 showed the highest AUC (0.656), indicating a moderate ability to distinguish between patients with and without residual pulmonary impairment. Other parameters with comparable performance included FEV_1_ (AUC = 0.633), DLCO% (AUC = 0.621), and MEF25 (AUC = 0.615).

A comprehensive evaluation of pulmonary function in respiratory diseases, including post-COVID-19 sequelae, requires multiple measurements such as spirometry (FVC, FEV_1_, MEF), diffusion capacity (DLCO, KCO), and peripheral oxygen saturation (SpO_2_).

### 3.3. Characterization of Pulmonary Function in Post-COVID-19 Patients (2021–2022) Using Principal Component Analysis (PCA)

To explore the underlying structure of these measurements and to group them into coherent functional domains, principal component analysis (PCA) with Varimax rotation was performed on a sample of 97 post-COVID-19 patients. Standardized clinical data were used according to ATS/ERS (2019) recommendations for spirometry and DLCO [22,25].

**Figure 3:**
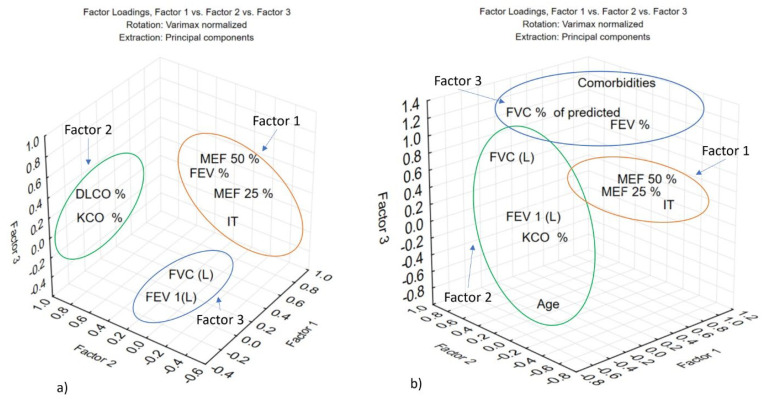
3D representation of Varimax-rotated factor loadings for the three principal components in the assessment of pulmonary function in PCS patients investigated in 2021 (**a**) and 2022 (**b**).

The PCA applied the Kaiser criterion (eigenvalue > 1) and Varimax rotation to identify meaningful factors. In the cohort investigated in 2021, three factors emerged as relevant to the clinical dataset of PCS patients (Table 4, Figure 3a).

Factor 1 explained 38.6% of the total variance and was primarily associated with MEF50%, MEF25%, IT, and FEV_1_%. Factor 2 accounted for 19% of the variance, encompassing KCO%, DLCO%, and DLCO, while Factor 3 represented 11.3% of the total variance and included FVC (L) and FEV_1_ (L).

Figure 3a shows the distribution of the variables within the three-factor space, providing a clear visualization of the functional domains extracted in the assessment of post-COVID-19 pulmonary function in 2021.

PCA with Varimax rotation applied to clinical and respiratory data from PCS patients in 2022 identified three significant factors explaining 69.64% of the total variance. Factor 1 (35.28%) reflects airway obstruction, with high loadings for IT, MEF50% and MEF25%. Factor 2 (21.96%) includes FVC, KCO% and FEV_1_ (L) with a negative contribution from age. (Supplementary Material) Factor 3 (12.40%) integrates FVC (%) and FEV_1_ (%) inversely related to comorbidities, acute COVID-19 severity, and persistent symptoms. Figure 3b shows the factor loadings, illustrating how variables cluster according to their contribution to each factor.

## 4. Discussion

The study aims to characterize pulmonary impairment by analyzing respiratory functional parameters and persistent symptoms in patients from the North region of Moldova (Romania) diagnosed with PCS between 2021 and 2022. It highlights the persistence of impaired pulmonary function in PCS patients, depending on the time elapsed since the acute infection and on the severity of the initial disease. Clinical analysis of spirometric and diffusion parameters demonstrated that FVC and FEV_1_, although generally close to normal limits, showed significant decreases in the first months post-infection, especially in patients with severe forms; the trend towards FVC recovery continued up to the 9–12-month interval *p* ˂ 0.05). These statistical differences suggest that, although partial recovery is possible, respiratory impairment may persist and requires long-term monitoring [26,27,28].

The prevalence of symptoms such as physical asthenia and dyspnea, correlated with the severity of the initial disease, underscores the clinical relevance of objective parameters in interpreting post-infection functional status. Accordingly, the increase in the proportion of patients with moderate forms at 9–12 months (72%) corresponds to significant decreases in FVC and DLCO, highlighting the link between initial severity and persistent respiratory impairment. These findings are consistent with recent studies showing that pulmonary sequelae may persist even 12 months after SARS-CoV-2 infection, particularly in patients with severe disease or with cardiovascular and metabolic comorbidities [29,30].

In addition, other studies highlight that pulmonary function abnormalities, such as decreased DLCO, are common and may indicate underlying interstitial damage [31].

The ROC analysis revealed that the parameters TLC, AV, and KCO demonstrate the highest predictive value for identifying post-COVID-19 pulmonary impairment (AUC > 0.80), suggesting their usefulness in standardized lung functional evaluation. In contrast, the parameters MEF50 and DLCO showed lower predictive capacity, indicating that not all traditional measurements provide the same clinical relevance and that the selection of monitoring parameters should be made carefully, in accordance with ATS/ERS recommendations [32]. Moreover, the results of the ROC curve analysis in this study suggest that pulmonary functional parameters, particularly MEF50 and FEV_1_, may provide a moderate estimate of post-COVID-19 pulmonary impairment; however, their accuracy remains limited. Therefore, it is necessary to employ approaches based on combined models that integrate multiple functional parameters to achieve a more robust and sensitive assessment of impairment in post-COVID-19 syndrome [33].

The results of the patients evaluated in 2022 (Figure 2b) showed that parameters such as FVC (AUC = 0.582), AV (0.575), TLC (0.589), and KCO% (0.566) exhibited low predictive value, suggesting a limited individual discriminative capacity. Although these values do not reach the threshold of statistical significance for accuracy (AUC ≥ 0.7), they may still indicate potential clinical usefulness in identifying pulmonary functional impairment [7].

The principal component analysis (PCA) enabled the identification of three distinct factors integrating spirometric parameters, diffusion indices, and the Tiffeneau index, accounting for 69.6% of the total data variance. The first factor reflects an obstructive dysfunction, consistent with airway impairment frequently observed in post-COVID-19 syndrome, particularly among patients with a history of smoking or pre-existing respiratory comorbidities [34]. These results are consistent with recent literature showing that post-COVID-19 patients may exhibit persistent obstructive ventilatory dysfunction, associated with alterations in MEF and the FEV_1_/FVC ratio [35,36].

The second factor suggests that this pattern is indicative of a restrictive impairment, frequently associated with residual parenchymal lesions or post-COVID-19 pulmonary fibrosis. A reduction in FVC, in the absence of obstruction, has been reported in multiple longitudinal studies, particularly among patients who experienced moderate or severe forms of the disease [15,37].

The association with age supports the hypothesis that lung function declines with aging, and elderly patients are more susceptible to developing a restrictive pattern in the post-viral period [38].

The third factor may be interpreted as an indicator of the systemic impact of the infection and the overall functional status of the post-COVID-19 patient. Recent studies have shown that the severity of the acute COVID-19 episode, as well as the presence of comorbidities (hypertension, obesity, diabetes mellitus), are predictors of persistent reductions in lung function parameters [39,40].

The comparative factor analysis between 2021 and 2022 highlights an interesting evolution of the latent clinical profile that may characterize post-COVID-19 lung dysfunction. Consistently, Factor 1 was dominated by spirometric parameters of the distal airways (MEF25%, MEF50%, and the Tiffeneau index), indicating persistent bronchial obstructive impairment. This profile is well documented in the literature, particularly in studies describing post-COVID-19 syndrome with peripheral ventilatory involvement [9,41].

In contrast, alveolar-capillary diffusion parameters (DLCO, KCO), which constituted a distinct dimension in 2021, became more dispersed across factors in 2022, suggesting increased heterogeneity of interstitial impairment. Longitudinal studies indicate that although DLCO is affected in over 40% of patients with moderate and severe forms during the acute phase, complete recovery is variable, and a significant proportion remain symptomatic and with subnormal values after 6–12 months [15,33,42].

This evolution may reflect the influence of multiple factors, including the severity of the acute form, the presence of comorbidities, and the individual immune response (vaccination, reinfection). It was also observed that Factor 3, weakly expressed in 2021, became more clearly defined in 2022 and comprised variables reflecting clinical severity (FEV_1_%, predicted FVC%) and the presence of comorbidities. This new structure suggests that, as the pandemic evolved, the post-COVID-19 functional profile began to more strongly reflect the interaction between pulmonary impairment and pre-existing systemic conditions [39,40,43].

Post-COVID-19 lung dysfunction has been observed to persist even 1–2 years after infection, particularly among patients who experienced severe disease. DLCO remains the most sensitive long-term affected parameter, indicating possible interstitial involvement. Continuous pulmonary function monitoring and inclusion of patients in respiratory rehabilitation programs are essential to prevent chronic complications. The study results highlight a pattern of slow but progressive pulmonary functional recovery in patients with post-COVID-19 syndrome, observed in both the 2021 and 2022 cohorts. The significant increase in FVC values between 3 and 9 months post-infection (*p* = 0.027), as well as between 9 and 18 months (*p* = 0.031), may suggest an ongoing process of reversibility of the restrictive dysfunction associated with inflammation and pulmonary parenchymal remodeling in the post-viral phase [28,44].

## 5. Conclusions

This study highlights the persistence of pulmonary function impairment up to 12 months after SARS-CoV-2 infection, particularly in patients with severe disease and comorbidities. Although partial recovery was observed, complete normalization of functional parameters was uncommon. TLC, AV, and KCO were identified as the most reliable predictors of post-COVID-19 lung dysfunction (AUC > 0.80), whereas MEF50 and FEV_1_ showed lower predictive value. Principal component analysis revealed three functional patterns—obstructive, restrictive, and mixed—reflecting the multifactorial nature of lung function impairment. These findings underscore the need for long-term respiratory monitoring and inclusion of patients in individualized rehabilitation programs. Standardized assessment of diffusion parameters and comprehensive spirometry is essential to optimize management and prevent chronic post-COVID-19 respiratory sequelae. Adopting standardized evaluation protocols, as recommended by ATS/ERS, may enhance early detection of chronic lung dysfunction and guide targeted therapeutic interventions.

### Limitations

This study has several important limitations. The retrospective design and the lack of pre-COVID-19 lung function data limit the clear interpretation of post-infection changes. The definition of post-COVID-19 syndrome is not standardized, which may lead to misclassification of patients. The study was conducted at a single center, limiting the generalizability of the findings. Additionally, the principal component analysis depends on the selected variables and should be interpreted alongside clinical evaluation. Prospective multicenter studies are needed to validate these results.

## Figures and Tables

**Table 1:** Demographic and clinical characteristics of patients with post-COVID-19 syndrome evaluated in 2021.

Demographic Data of Patients from 2021	1–3 Months After the Acute Episode		4–7 Months After the Acute Episode		9–12 Months After the Acute Episode		
	***n*** ** = 36**		***n*** ** = 18**		***n*** ** = 11**		***p***
Age(Years) Mean(SD) Min–Max%	52.22 (12.73) 26–76		54.78 (15.79) 27–79		53.50 (17.31) 27–77		0.41
Urban/rural area (%)	86/14		70/30		90/10		-
Sex (female/male)%	61/39		55/45		70/30		-
Smoking history: smokers/non-smokers/former smokers/% NR *	17/42/11/30		10/50/10/30		10/45/27/18		-
COVID-19 Severity: Mild/moderate/severe%	64/36		55/35/10		28/72		˂0.05
Comorbidities%							˂0.0001
Hypertension%	11		15		36%		˂0.0001
Diabetes mellitus%	5		10		18%		˂0.0001
Respiratory disorders%	14		15		2%		˂0.0001
Obesity%	5		10		5%		˂0.0005
Thyroid disease%	11		-		-		
The most common post-COVID-19 symptoms:							
Physical fatigue%	69		40		60		˂0.005
Cough%	22		35		30		˂0.005
Dyspnea%	50		40		40		˂0.005
**Pulmonary function parameters**	**Mean (Stdev)**	**Min–Max**	**Mean (Stdev)**	**Min–Max**	**Mean (Stdev)**	**Min–Max**	
SpO_2_%	96.86 (1.53)	93–99	96.5 (1.12)	95–99	96.5 (1.11)	95–99	0.189
FVC% of predicted	101.58 (12.03)	72.5–129	103.5 (15.36)	74.9–133	94.6 (8.80)	83.6–110	˂0.05
FEV1%	104.0 (12.57)	74.7–129	108.2 (16.7)	78.4–147	96.28 (11.28)	72.9–115	˂0.01
IT	84.05 (5.09)	76–97.4	85.0 (4.39)	75.8–90.1	85.05 (5.68)	71.1–91.9	0.143
MEF 50%	100.66 (27.89)	58.7–175	105.1 (28.85)	59–158	110.15 (36.31)	34.1–162	˂0.005
MEF 25%	88.38 (39.68)	32.1–204	92.18 (28.18)	40.7–129	96.22 (22.37)	68.5–130	0.261
DLCO%	88.44 (16.33)	57–122	92.18 (19.02)	64–125	82.9 (10.05)	72–104	˂0.001
KCO%	96.82 (13.76)	69–137	96.7 (14.431)	76–119	100.8 (10.41)	83–118	˂0.01

* Unspecified value.

**Table 2:** Demographic and clinical characteristics of patients with post-COVID syndrome evaluated in 2022.

Demographic Data of Patients from 2022	3–6 Months After the Acute Episode		7–12 Months After the Acute Episode		14–22 Months After the Acute Episode		*p*
	***n* = 19**		***n* = 6**		***n* = 7**		
**Age (years) Mean (SD) Min–Max**	59.55 (13.49) 22–77		65 (10.58) 54–81		64.28 (8.46) 46–85		0.53
**Urban/rural area (%)**	89/11		100/0		57/43		-
**Sex (female/male) (%)**	53/47		50/50		71/29		-
Smoking history-smokers/non-smoker/former-smoker/UV * (%)	18/52/15/15		-/38/35/27		14/29/29/28		-
COVID-19 Severity: Mild/moderate/severe	43/42/15		66/34/-		29/71/-		-
**Comorbidities (%)**							0.85
Diabetes mellitus%	20		-		10		-
Hypertension%	35		14%		30		-
Neoplasma%	10		-		-		-
CIC% Thyroid disease%	- 11		14 -		- -		-
**The most common post-COVID-19 symptoms:**							-
Physical fatigue%	45		28		40		-
Caugh%	20		10		50		-
Dyspnea%	45		43		60		-
**Pulmonary function parameters**	**Mean (Stdev)**	**Min–Max**	**Mean (Stdev)**	**Min–Max**	Mean (Stdev)	Min–Max	
SpO_2_%	96.61 (2.29)	90–99	97.83 (0.69)	96–99	96.86 (1.46)	87–99	0.14
FVC % of predicted	95.57 (14.36)	66.7–117	117.5 (17.73)	86.1–145	100.9 (3.62)	51.8–105	˂0.05
FEV1%	97.2 (20.41)	43.9–128	116.97 (27.30)	73.7–162	90.81 (21.10)	37.3–108	˂0.05
IT	85.7 (11.32)	47.1–98.1	83.78 (8.34)	64.7–92.7	79.93 (8.12)	61.6–88.7	0.89
MEF 50%	100.08 (23.01)	12.4–135	109.9 (38.38)	41.4–153	82.71 (21.06)	40.10–105	
MEF 25%	92.59 (34.71)	16–166	92.8 (50.67)	33.5–172	70.77 (25.46)	30.2–110	0.076
DLCO%	83.78 (17.03)	45–120	91.40 (13.38)	72–110	79.40 (9.13)	68–92	0.34
KCO%	96.44 (19.09)	68–134	93.10 (16.15)	75–120	88.20 (12.81)	75–109	0.68

* Unspecified value, CIC—Chronic ischemic heart disease.

**Table 3:** Statistical comparison of pulmonary function parameters between patients with mild and moderate post-COVID-19 disease (2021–2022).

Mann–Whitney U Test Post-COVID 2021/2022 by Severity Form/COVID-19 Marked Tests are Significant at *p* ˂ 0.05									
PCS (2021)	Rank Sum	Rank Sum	U	Z	*p* Value	Z	*p* Value	Valid N Mild Form	Valid N Moderate Form
Age	981.5	1163.5	240.5	−3.62	0.0003	−3.62	0.0003	36	27
Time since the acute episode	1105.0	1040.0	364.0	−1.98	0.0481	−2.02	0.0429	36	27
FVC% from predicted	1501.5	643.5	265.5	3.29	0.0010	3.29	0.0010	36	27
FEV1%	1430.0	715.0	337.0	2.34	0.0195	2.34	0.0194	36	27
DLCO%	1491.5	653.5	275.5	3.15	0.0016	3.16	0.0016	36	27
KCO%	1485.5	659.5	281.5	3.08	0.0021	3.08	0.0021	36	27
**PCS (2022)**									
DLCO%	409.0	186.0	50.0	3.23	0.0013	3.23	0.0012	18	14
KCO%	401.0	194.0	58.0	2.95	0.0032	2.96	0.0031	18	14

**Table 4:** Percentage contribution of principal components in PCA for PCS patients (2021–2022).

Eigenvalues (**post-COVID 2021.sta**) Extraction: Principal components				
	Eigenvalue	% Total	Cumulative	Cumulative
Factor 1	6.179288	38.62055	6.17929	38.62055
Factor 2	3.038773	18.99233	9.21806	57.61288
Factor 3	1.800743	11.25464	11.01880	68.86752
Eigenvalues (**post-COVID/2022.sta**) Extraction: Principal components				
	Eigenvalue	% Total	Cumulative	Cumulative
Factor 1	6.351194	35.28441	6.35119	35.28441
Factor 2	3.952570	21.95872	10.30376	57.24314
Factor 3	2.231929	12.39961	12.53569	69.64274

## Data Availability

Data are contained within the article.

## Supplementary Materials

The following supporting information can be downloaded at: https://www.mdpi.com/article/10.3390/jcm15020717/s1, Table S1. Factor Loadings (Varimax normalized) (data PCS 2021.sta) Extraction: Principal components.
