# Supplementary material for: Impact of COVID-19 on Respiratory Function: A Post-Recovery Comparative Assessment
Source: J Clin Med. 2026 Jan 15;15(2):717. doi: 10.3390/jcm15020717 (PMC12842235; doi:10.3390/jcm15020717)
Supplement: Supplementary file 1 [file jcm-15-00717-s001.zip › jcm-4026460-supplementary.pdf]

Table S1. Factor Loadings (Varimax normalized) (data PCS 2021.sta) Extraction: Principal components

	Factor1	Factor2	Factor3
Age	-0.089	-0.270	-0.659
Time since the acute episode	0.110	0.030	-0.360
SpO2	-0.001	-0.047	0.568
FVC (L)	0.302	0.235	0.856
FVC % from predicted	0.408	-0.009	0.451
FEV (L)	0.456	0.230	0.801
FEV 1 %	0.724	-0.009	0.331
IT	0.841	0.048	-0.274
MEF 50 %	0.929	-0.020	-0.023
MEF 25 %	0.907	0.010	-0.024
DLCO (mmol/(min*kPa))	0.165	0.589	0.643
DLCO %	-0.023	0.794	0.204
KCO %	0.061	0.884	-0.094

Factor Loadings (Varimax normalized) (data PCS 2022.sta) Extraction: Principal components

	Factor1	Factor2	Factor3
Age	0.077	-0.851	0.143
History of smoking	-0.541	0.263	0.031
COVID-19 severity	0.246	0.332	-0.563
Time after the acute episode	-0.286	0.016	-0.391
COVID-19 vaccination status	0.046	0.059	0.493
Comorbidities	0.244	-0.038	-0.787
Symptoms	0.507	0.224	-0.521
SpO2	0.367	0.423	0.336
FVC (L)	-0.295	0.884	-0.042
FVC % of predicted	0.222	0.039	0.862
FEV (L)	-0.099	0.856	-0.134
FEV %	0.541	-0.140	0.748
IT	0.928	-0.258	0.036
MEF 50 %	0.925	0.002	0.241
MEF 25 %	0.843	0.191	0.169
DLCO (mmol/(min*kPa))	-0.687	-0.022	0.098
DLCO %	-0.631	-0.209	0.579
KCO (mmol/min*kPa*L))	-0.378	-0.671	0.066
KCO %	-0.342	-0.777	-0.095
